# Accuracy of low‐dose proton CT image registration for pretreatment alignment verification in reference to planning proton CT

**DOI:** 10.1002/acm2.12565

**Published:** 2019-04-01

**Authors:** Roberto Cassetta, Pierluigi Piersimoni, Marco Riboldi, Valentina Giacometti, Vladmir Bashkirov, Guido Baroni, Caesar Ordonez, George Coutrakon, Reinhard Schulte

**Affiliations:** ^1^ Department of Electronics, Information and Bioengineering (DEIB) Politecnico di Milano University 20133 Milan Italy; ^2^ Department of Biomedical Physics in Radiation Oncology German Cancer Research Center 69120 Heidelberg Germany; ^3^ Department of Medical Physics Ludwig‐Maximilians‐Universität München 85748 Garching Germany; ^4^ Radiation Physics University of Wollongong Wollongong NSW 2522 Australia; ^5^ Department of Basic Sciences Loma Linda University Loma Linda CA 92350 USA; ^6^ Center for Research Computing and Data Northern Illinois University DeKalb IL 60115 USA; ^7^ Department of Physics Northern Illinois University DeKalb IL 60115 USA

**Keywords:** deformable image registration, image reconstruction, proton CT, rigid image registration

## Abstract

**Purpose:**

Proton CT (pCT) has the ability to reduce inherent uncertainties in proton treatment by directly measuring the relative proton stopping power with respect to water, thereby avoiding the uncertain conversion of X‐ray CT Hounsfield unit to relative stopping power and the deleterious effect of X‐ ray CT artifacts. The purpose of this work was to further evaluate the potential of pCT for pretreatment positioning using experimental pCT data of a head phantom.

**Methods:**

The performance of a 3D image registration algorithm was tested with pCT reconstructions of a pediatric head phantom. A planning pCT simulation scan of the phantom was obtained with 200 MeV protons and reconstructed with a 3D filtered back projection (FBP) algorithm followed by iterative reconstruction and a representative pretreatment pCT scan was reconstructed with FBP only to save reconstruction time. The pretreatment pCT scan was rigidly transformed by prescribing random errors with six degrees of freedom or deformed by the deformation field derived from a head and neck cancer patient to the pretreatment pCT reconstruction, respectively. After applying the rigid or deformable image registration algorithm to retrieve the original pCT image before transformation, the accuracy of the registration was assessed. To simulate very low‐dose imaging for patient setup, the proton CT images were reconstructed with 100%, 50%, 25%, and 12.5% of the total number of histories of the original planning pCT simulation scan, respectively.

**Results:**

The residual errors in image registration were lower than 1 mm and 1° of magnitude regardless of the anatomic directions and imaging dose. The mean residual errors ranges found for rigid image registration were from −0.29 ± 0.09 to 0.51 ± 0.50 mm for translations and from −0.05 ± 0.13 to 0.08 ± 0.08 degrees for rotations. The percentages of sub‐millimetric errors found, for deformable image registration, were between 63.5% and 100%.

**Conclusion:**

This experimental head phantom study demonstrated the potential of low‐dose pCT imaging for 3D image registration. Further work is needed to confirm the value pCT for pretreatment image‐guided proton therapy.

## 1 INTRODUCTION

Proton therapy provides superior dose distributions in the low to intermediate dose range compared to photon therapy, which may lead to improved outcomes for some types of cancer and reduced side effects.^1^, ^2^, ^3^ Uncertainties in patient positioning and beam range as well as internal changes of tumor and patient anatomy could, however, compromise treatment effectiveness.^4^ Therefore, efforts to develop and improve treatment planning accuracy and image guidance for proton therapy are ongoing.^5^, ^6^ Currently, for treatment planning in proton therapy, an X‐ray CT dataset of the patient is acquired and Hounsfield units of the scan are converted to relative stopping power (RSP). This conversion is one important source for range uncertainties, which are typically estimated on the order of 3–5% of the planned proton range.^7^ Replacing X‐ray planning CT with proton CT (pCT) planning CT simulations with individual proton tracking during the scan has been proposed as a low‐dose method to reduce this planning uncertainty; pretreatment pCT would also provide a method for pretreatment verification of correct patient setup and RSP distribution. This method is currently in the preclinical stage of its development.^8^, ^9^, ^10^

The potential advantages of pCT for image guidance in the treatment room are several‐fold: (a) There is a dose advantage compared to X‐ray cone‐beam CT (CBCT) and (b) there is absence of artifacts often present in X‐ray CT based reconstructions; (c) using the same radiation source would allow imaging the patient immediately before treatment in the treatment position; (D) finally, the largest advantage of pCT would be that it could detect range errors before treatment in addition to serving as a low‐dose alignment technique that could replace CBCT. Therefore, daily 3D verification of patient alignment relative to the proton beam and confirmation that the RSP distribution on the beam path has not changed from the original treatment plan could be a valuable development for proton therapy, as it would allow better treatment accuracy and narrower margins, especially for hypofractionated treatment schedules.

Proton CT based on individual particle tracking utilizes position and direction information of the protons before and after the patient and measures the energy deposited by protons that traversed the object in a scintillator. Using this information from many protons, typically of the order of 100 protons per cm^2^, coming in from many discrete or continuous directions, one can reconstruct the distribution of the RSP with sufficient spatial resolution.^10^

One of the challenges in proton imaging is the degraded spatial resolution due to multiple Coulomb scattering (MCS) inside the imaged object. To improve the resolution, several most likely path (MLP) formulations have been proposed and are used in pCT image reconstruction.^11^, ^12^, ^13^ Iterative algorithms can then be used to reconstruct 3D pCT images from radiological projections. With these developments, including fast parallel processing of the acquired pCT data, a clinical setting for pCT appears feasible.

The purpose of this study was to evaluate the performance of pCT for pretreatment image guidance using rigid and deformable image registration algorithms. A high‐quality planning CT simulation scan was created by experimentally scanning a head phantom and a reconstruction algorithm using all available proton histories and FBP as initial iterate followed by an iterative reconstruction algorithm. In addition, pretreatment pCT scans were generated for different imaging doses by selecting different number of proton histories entering the reconstruction and using only fast FBP as the reconstruction method. These pretreatment scans were then rigidly transformed by prescribing random 3D errors (rotations and translations) to simulate random alignment errors. The study endpoint was the accuracy of the image registration algorithm in recovering the original planning pCT simulation scan as a function of the different imaging dose levels. In the second part of the study, a deformation field derived from a real patient was applied (a) to the original planning pCT study to simulate a deformed pretreatment pCT using all histories and FPB plus iterative reconstruction that could be used for replanning and (b) to the FBP‐only reconstructed preplanning pCT scans to simulate the accuracy of registration in the presence of deformation and at different doses.

## 2 METHODS

### 2.A Proton CT scanner and study design

**Figure 1:**
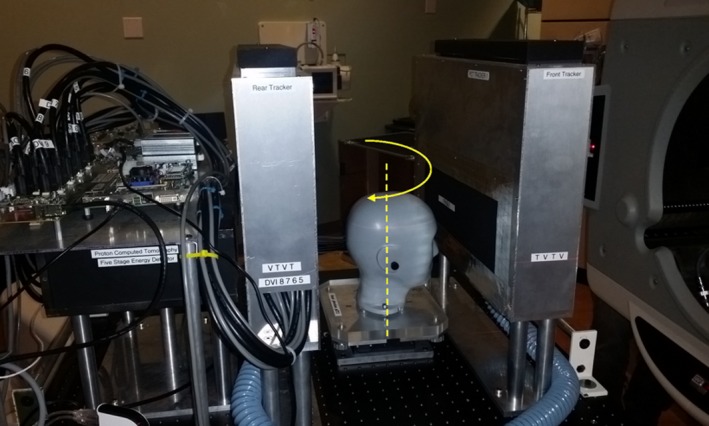
Scanner built by the pCT collaboration. The scanner and the head phantom are shown in the scanning position on the horizontal proton beam line. The proton beam traverses the scanner space from right to left while the phantom rotates in discrete steps or continuously.

The prototype pCT scanner, built by the pCT collaboration was used for this work (Fig. 1). It consists of a front and rear tracker system used to extrapolate the proton path before and after the object and a multi‐stage scintillator (MSS) allowing the measurement of the proton residual energy and converting it to water equivalent path length (WEPL).^14^ The trackers comprise four planes of position‐sensitive Si‐strip detectors oriented in either vertical or horizontal direction. Per tracker, the proton location is registered in two locations allowing a direction vector to be reconstructed. The sensitive tracking area is 36 cm in horizontal direction and 9 cm in vertical direction. For a complete scan of the head phantom, two single 360‐degree scans were performed with a vertical shift of 8 cm between the two scans. The scanner was installed on the clinical horizontal proton beam line at the Northwestern Medicine Chicago Proton Center, Warrenville, IL and tested with an anthropomorphic head phantom (HN715, CIRS), which was positioned on a rotating stage. A single pCT scan for treatment planning takes 6 min, acquiring about 360 M proton histories (before data cuts) during 6 full rotations of the stage at 1 rpm and using 200 MeV protons (range of 26 cm in water). One should note that while 1 rpm would match the standard rotational speed of proton gantry, the current prototype pCT scanner is this limited to registering about 1 million protons per second. In a future implementation, the pCT scanner acquisition rate will be increased by a factor 2‐3, making it possible to acquire the scan in 2‐3 rotations at 1 rpm. The tracker and MSS data of individual protons were read out by a custom high‐speed data acquisition (DAQ) system, capable of handling data rates on the order of 1 million protons/sec.^8^, ^10^ To determine WEPL, the MSS detector response was calibrated using a step‐phantom of known water‐equivalent thickness.^14^ For high‐fidelity treatment planning pCT simulations, a 3D filtered back projection (FBP) algorithm was employed initially to determine the object boundaries; subsequently it was used as the first iterate for the subsequent iterative image reconstruction. The reconstruction for the planning pCT simulation was achieved in under 7 min with high‐performance computing.^15^ The FBP without further refinements of RSP values by iterative reconstruction was obtained in under 1 min, and was used for image registration in a pretreatment situation (pretreatment pCT).

Image registration (IR) of the pretreatment pCT scan to the original planning pCT simulation was used to determine the spatial transform for the alignment of the head phantom after the study had been intentionally been transformed by a random 3D vector and three random rotations about the cardinal axes. A rigid IR procedure was used for finding three translations and rotation angles that realigned the pretreatment pCT to the original planning pCT simulation.

### 2.B Experimental pCT data

**Figure 2:**
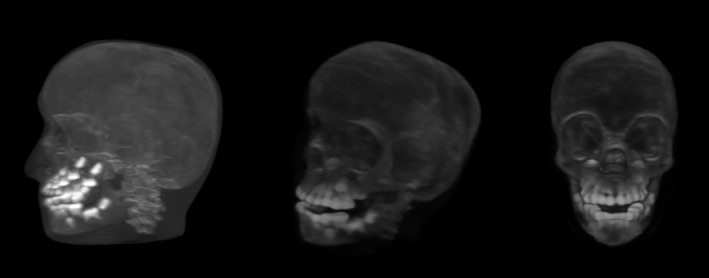
3D‐volumentric pCT reconstruction of the pediatric head phantom

**Figure 3:**
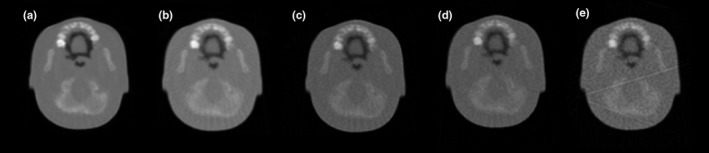
Different image types used in this study: (a) planning pCT simulation, (b) FBP_100_, (c) FBP_50_, (d) FBP_25_, (e) FBP_12.5_.

For the planning pCT simulation scan, 90 projections of the pediatric head phantom (model 715‐HN, CIRS) were obtained with the prototype proton CT scanner.^10^ The pCT data processing and image reconstruction steps are as follows. The acquired pCT data (histories) are checked for completeness and consistency and then converted to tracker coordinates and MSS response values. A pre‐scan WEPL calibration scan with a calibration object is used to construct a calibrated relationship to convert MSS responses to WEPL values. Since the active tracker area is 9 cm in cranio‐caudal direction, two successive scans of the head phantom were obtained with a longitudinal shift of the phantom of about 8 cm between the two scans. For each scan, a total number of about 200 M protons entered the reconstruction process. For the planning pCT simulation scan the 3D FBP algorithm was used as the initial step producing an initial approximate solution followed by five iterations of the total‐variation superiorization diagonally relaxed projections (TVS‐DROP) algorithm described and used for pCT reconstruction previously^16^ (Fig. 3). These reconstructed images were then combined into a 3D DICOM image (Fig. 2) with a voxel size of 0.58, 0.58 and 1.25 mm for right‐left (RL), anteroposterior (AP), and cranio‐caudal (CC) direction, respectively.

For the pretreatment pCT scans, the 3D registration algorithm capability of successful patient positioning with very low‐dose images was evaluated. Image reconstructions consisting of FBP only were performed with a consecutively reduced number of protons using 100%, 50%, 25%, and 12.5% of the dose of the planning pCT simulation scan. The dose to the head phantom corresponding to 100% was estimated by scanning a 16‐cm acrylic head phantom (Catphan model CTP 554) with a PTW Farmer ionization chamber inserted at its center using a similar total number of proton triggers and scanning time. The dose to the phantom center was measured to be 1.45 ± 0.3 mGy (mean value and standard deviation of two independent measurements). In the remainder of this paper, the regular and low‐dose reconstructions will be referred as FBP_100_, FBP_50_, FBP_25_, and FBP_12.5_, respectively. These low‐dose pretreatment pCT scans were then further modified to simulate random setup errors and a deformation from the original scan as described below. A visual comparison of representative pretreatment pCT images used in this study can be seen in Fig. 3. The signal‐to‐noise ratio (SNR) of the different reconstructions was obtained by dividing the average intensity from a circular region inside the phantom in the pCT images by the standard deviation of background values. The SNR ratios for each type of image reconstruction were: 8.12, 5.86, 5.00, 4.99, and 4.02 for the planning pCT, FBP_100_, FBP_50_, FBP_25_, and FBP_12.5_, respectively.

### 2.C Rigid image registration algorithm

A 3D algorithm for rigid image registration was developed based on the Insight Segmentation and Registration Toolkit (ITK) open software library.^17^ Mattes mutual information,^18^ often applied for multi‐modality images, was used as the similarity metric. The intrinsic advantage of this method is image rescaling when the discrete density function is built.^19^ This metric tends to map homogeneous regions from the moving image into homogeneous regions of the fixed image. The mutual information is a statistical comparison of the images based on their intensity distribution and shows robustness even with image noise and heterogeneous image superposition. A regular‐step gradient descent optimization method was used as the optimizer for the rigid image registration, in order to minimize the metric expression until the termination criterion set by the user, that is, a minimum step length (0.001) or 200 iterations, was reached. The main features of the algorithm are summarized in Table 1.

**Table 1:** Rigid registration algorithm features

Component	Component name	Notes
Optimizer	Regular step gradient descent optimizer	Parameters are set based on “time x precision” tradeoff
Metric	Mattes mutual information	
Transform	Euler 3D transform	
Transform Initializer	Centered transform initializer	The computation of the center of mass decreased IR time significantly

### 2.D Deformable image registration algorithm

A custom algorithm was written using the ITK open software library to handle the deformable image registration. The metric used in this algorithm was the same as that used for the rigid registration (Mattes Mutual Information). The limited‐memory Broyden–Fletcher–Goldfarb–Shanno (LBFGS)^20^ method was used as the optimizer for deformable image registration, in order to minimize the metric expression until termination criteria, e.g., the cost function convergence factor or gradient tolerance, are reached. The main components of the developed algorithm are presented in Table 2.

**Table 2:** Deformable registration algorithm features

Component	Component name	Notes
Optimizer	LBFGS	Parameters are set based on “time × precision” tradeoff
Metric	Mattes mutual information	
Interpolator	B‐Spline	
Filter	Histogram matching	For multi‐modality image registration

LBFGS, limited‐memory Broyden–Fletcher–Goldfarb–Shanno

### 2.E Performance evaluation

To evaluate the accuracy of rigid registration using pCT scans, 10 random 6‐degree‐of‐freedom (DOF) transformations (translation and rotation) were created using orthogonal sampling^21^ and applied to each set of images to be registered. The images were then resampled using the Lanczos filter in the Amira 3D software platform (version 5.3.3, FEI Visualization Sciences Group). The transformations were within the clinically meaningful range of ±3 mm for translations and ±5° for rotations. Ten different setup misalignments were thus simulated by using this procedure at all different levels of pCT images dose used in this study. After registering each pair of images, the residual distance between known transformation and suggested corrections were calculated as a measure of the registration error.

The registration procedures were carried out on a notebook with Intel Core i7‐4710HQ 2.50 GHz processor and 16.0 GB installed memory: the mean computation time for the rigid registration was 2.5 min. By changing the stopping criteria or reducing the image size, the user can improve accuracy or reduce the computational time. The parameters can be changed; therefore, it is possible to decide how much similarity is enough to stop the IR process. In our case, the minimum step length of 0.001 (as suggested on ITK documentation examples) was maintained and was found to be sufficient to reach clinical accuracy of the procedure in an acceptable time.

**Figure 4:**
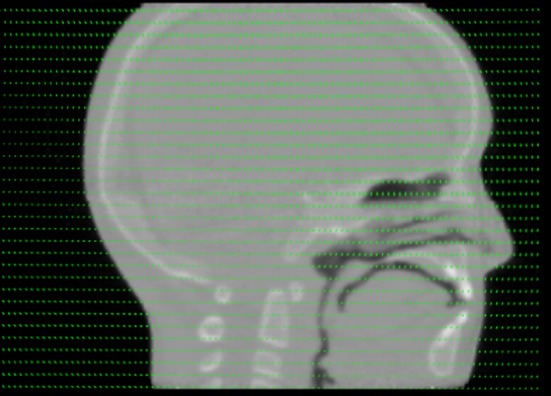
Sagittal mid‐plane reconstruction after a patient‐specific deformation field was applied to the planning pCT simulation study.

To evaluate the accuracy of deformable registration using pCT scans, a realistic deformation field was obtained from the planning X‐ray CT and subsequent cone beam CT of a real patient treated with radiotherapy for head and neck cancer. The deformation field was then applied (a) to the original planning CT simulation study to represent a high quality pCT study of a realistically deformed phantom at the time of treatment, (Fig. 4), and (b) to the pCT_FBP_ images reconstructed at 100%, 50%, 25%, and 12.5% of the total dose of the planning pCT scan to simulate fast/low dose pretreatment pCT image reconstructions. The individual image sets were then deformably registered to the original planning CT simulation study.

After registering each pair of images, the scale invariant feature transform (SIFT)^22^ was used to extract features and to calculate the residual 3D distance between corresponding landmarks to numerically assess the quality of the registration.^23^

The deformable image registration (DIR) procedures were carried out on the same notebook as rigid transformation procedures: the mean computational time was 6 min in this case. The user can improve accuracy or reduce the computational time by changing optimizer settings such as cost function convergence factor, projected gradient tolerance, maximum number of evaluation and corrections, number of iterations, and number of grid nodes in one dimension. The convergence factor and gradient tolerance values were kept as those suggested by the ITK manual example. The number of evaluations was increased if further corrections were deemed necessary. A step to cache the B‐Spline weights and indexes related to each sample used to compute the metric was implemented. This made the DIR faster while allocating more memory.

## 3 RESULTS

### 3.A Rigid registration

**Figure 5:**
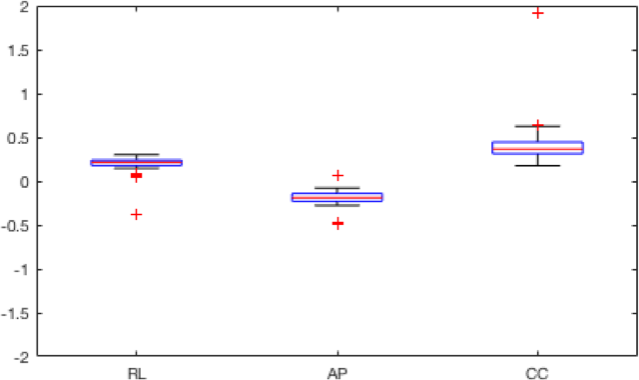
Boxplot for translation residuals after IR. The horizontal lines of the boxes represent the first and third quantile of the distribution, the center line corresponds to the median, and the lower and upper whiskers correspond to the minimum and maximum values, respectively unless outliers were present (marked with + symbol), which were defined as values 1.5× the inter‐quartile range below or above the first and third quartile values.

After the registration procedure, the differences between imposed errors and suggested corrections were calculated. The mean and standard deviation values of the residual distance for the 10 different simulated shifts for each IR modality are summarized in Table 3 for translation and in Table 4 for rotation. Translations (T) are expressed in millimeters and rotations (R) in degrees for RL, AP, and CC directions and axes, respectively. The residuals magnitudes found are similar, so they were grouped into anatomical directions and shown into box plots to illustrate their distribution on Figs. 4 and 5.

**Table 3:** Residual translational errors after rigid registration

Registration modality	T RL (mm)	T AP (mm)	T CC (mm)
pCT – FBP_100_	0.11 ± 0.18	−0.18 ± 0.13	0.51 ± 0.50
pCT – FBP_50_	0.22 ± 0.06	−0.23 ± 0.09	0.37 ± 0.12
pCT – FBP_25_	0.24 ± 0.04	−0.21 ± 0.05	0.35 ± 0.03
pCT – FBP_12.5_	0.17 ± 0.08	−0.14 ± 0.07	0.44 ± 0.13

**Table 4:** Residual rotational errors after rigid registration

Registration modality	R RL (deg)	R AP (deg)	R CC (deg)
pCT – FBP_100_	0.08 ± 0.08	0.04 ± 0.10	−0.00 ± 0.13
pCT – FBP_50_	0.04 ± 0.06	0.04 ± 0.10	−0.04 ± 0.13
pCT – FBP_25_	0.02 ± 0.04	0.04 ± 0.09	−0.05 ± 0.13
pCT – FBP_12.5_	0.04 ± 0.05	0.04 ± 0.08	−0.02 ± 0.13

### 3.B Deformable registration

**Figure 6:**
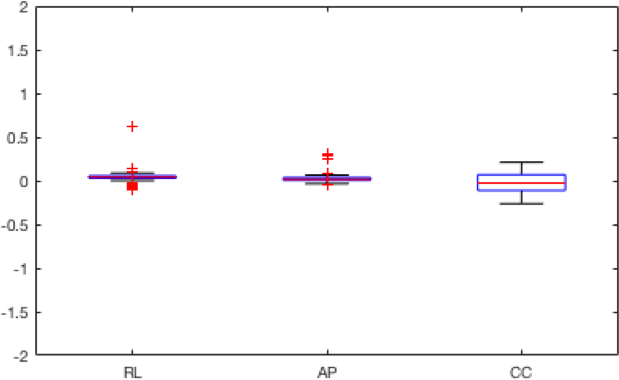
Boxplot for rotations residuals after IR. For further explanations, see legend of Fig. 5.

**Figure 7:**
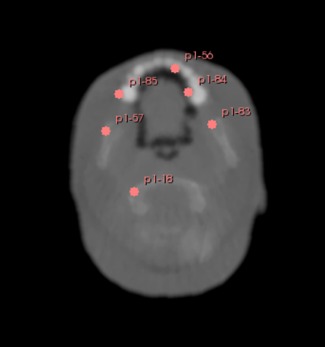
Examples of landmarks used for distance calculation between fixed and transformed image.

**Figure 8:**
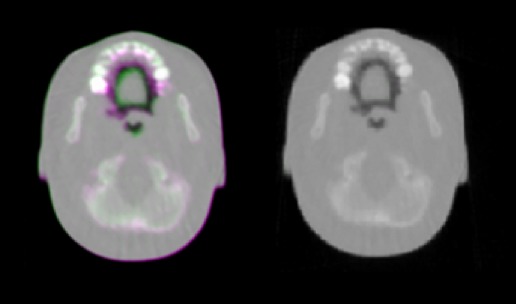
Left: Deformed pCT image overlaid with the original image; voxels with higher RSP values for the deformed image are shown in purple and those with higher RSP values for the original (fixed) image are shown in green. Right: The deformed image after application of DIR overlaid with the fixed image.

After the deformable registration procedure, on average, 44 corresponding markers between the fixed and the transformed image were identified using SIFT (Figs. 6 and 7) for the pCT images. The percentage of sub‐millimetric errors of the residual distance between landmarks calculated for each case after DIR are presented in Table 5. An example of images before and after DIR is presented in Fig. 8.

**Table 5:** Sub‐millimetric error distribution after DIR

Registration Pair	Percentage of sub‐millimetric errors
pCT – pCT	100
pCT – FBP_100_	63.5
pCT – FBP_50_	71.4
pCT – FBP_25_	64.4
pCT – FBP_12.5_	64.1

## 4 DISCUSSION

Image registration is an important aspect of image‐guided radiotherapy, and is particularly important for accurate proton therapy. In this work, we explored in an initial, admittedly limited experimental study, the use of a preclinical prototype pCT scanner for pretreatment alignment with a head phantom. Proton CT requires high‐energy protons to traverse the patients for imaging. At this point, the pCT method is limited to head and neck applications but is expected to also work for most patients in the thorax region; remaining body regions (pelvis and abdomen) would require energies in excess of 250 MeV, which are currently not clinically available, but should become available soon. For body scans, the use of helium ions would be more advantageous since it is less effected by MCS.

Two IR algorithms utilizing the ITK open software package were developed and tested for registration of experimental planning pCT simulation images of a pediatric head phantom. The experimental dataset used in this study came from transformed images generated from a single pCT acquisition, which limits the generalization of our findings to more realistic scenarios encountered with randomly repositioning of the patient. One could argue, however, that the selection of random data subsets for reduced‐dose reconstruction lessens the bias introduced by the correlation of the image pairs that were used for testing the accuracy of IR with pCT in this work. The actual performance of IR algorithm in the use of pCT for patient setup could, in principle, be confirmed through experimental measurements where images were acquired after changing the position of the phantom with the 6‐DOF patient positioner. This was not possible with the current experimental setup because the pCT scanner and head phantom platform were rigidly connected and mounted as one unit on the patient positioner. In the future, we are planning to implement an additional 6‐DOF mounting feature for the phantom that will allow independent translational and rotational misalignments relative to the treatment room coordinate system.

Nevertheless, the implemented study provided the quantification of expected performance in a controlled scenario, where the amount of rigid mismatch is known and the results are believed to be representative of the clinical situation with random variations in the position of a patient. The largest error found was 2 mm in the cranio‐caudal direction for the FBP_100_ images. The FBP reconstruction introduced some radial artefacts in certain anatomically heterogeneous regions of the images, not present in the planning pCT images due to additional iterative reconstruction. These features may have interfered with the DIR procedure and lead to systematic errors. However, these radiation artefacts were mostly masked by additional noise in the low‐dose FBP images and, therefore, the interference was not observed.

Low‐dose FBP‐only images, used for DIR with the planning pCT simulation, present larger SIFT‐detected errors even if visually the images seem almost perfectly aligned. These errors are mostly due to the lower quality of images, quantified by lower SNR associated with dose reduction, which would interfere in automatic feature detection, presenting up to 44% of the errors between 1 and 2 mm (same magnitude of pixel size in cranio‐caudal direction). By performing DIR between two planning pCT simulations, optimal alignment results were found.

Proton CT has the potential to be a useful tool for planning simulation and patient setup in proton therapy. Due to the ongoing developments in pCT imaging technology and reconstruction, which could meet clinical promptness requirements soon and precise RSP values, a pretreatment pCT (FBP + 5 iterations) could be acquired on daily basis for dose recalculation, aiming at ultimate treatment delivery effectiveness. For less sophisticated and faster plan adjustments, pCT_FBP_ images may be sufficient for plan adaptation with DIR. The next steps in this development is to increase the data rate of the pCT system working in tracking acquisition mode from currently 1.3 M protons per second to about 6 M protons, and eventually to 10 M protons per second as well as increasing the sensitive area to about 30 cm × 40 cm, thus allowing a single head pCT scan to be accomplished in 1.5 min or less. Ongoing pCT image reconstruction during DAQ is another topic of current interest and development.

Compared to an estimated standard CBCT head dose of 10 mGy; a dose of about 1.45 mGy^24^ for the full planning pCT simulation gives higher quality images with noteworthy dose reduction to the patient. Even for a histories reduction to 12.5% (~0.18 mGy), corresponding to about 55‐times dose reduction, the majority of residual errors were still found to have submillimeter magnitude. We feel that further decreasing dose is not required from a radiobiological standpoint. Proton CT images could be acquired on a daily basis for registration and dose recalculation, making pCT a very attractive modality for image guidance. Proton radiography (pRad) can also be acquired with the treatment gantry for patient alignment or patient‐specific RSP measurements to update, for example, the planning X‐ray CT calibration curve. The detectors described previously for the pCT scanner can be used for obtaining proton 2D projections to be used on a 2D‐3D registration. This a procedure analogous to the currently used method of registering X‐ray DRRs from the planning CT to two in‐room orthogonal X‐ray projections.

In summary, this was the first study of using pCT for planning and pretreatment patient alignment. Our study was limited to a single pCT study that was mathematically modified. The next step in this research will be to perform a more realistic study with an actually modified head phantom position, deformation, and changes in RSP values registered to an original pCT planning simulation scan.

## 5 CONCLUSION

This work demonstrated the potential of 3D head image registration based on proton CT for in‐room pretreatment verification. The developed algorithms for image registration can be accurate even at very low proton imaging doses. Nevertheless, the alignment could be influenced by image artifacts that were introduced by the fast filtered back projection reconstruction.

## CONFLICT OF INTERESTS

All authors declare that they have no conflict of interest.

## References

[1] Yock TI , Yeap BY , Ebb DH , et al. Long‐term toxic effects of proton radiotherapy for paediatric medulloblastoma: a phase 2 single‐arm study. Lancet Oncol. 2016;17:287–298. 26830377 10.1016/S1470-2045(15)00167-9

[2] Mendenhall NP , Hoppe BS , Nichols RC , et al. Five‐year outcomes from 3 prospective trials of image‐guided proton therapy for prostate cancer. Int J Radiat Oncol Biol Phys. 2014;88:596–602. 24521677 10.1016/j.ijrobp.2013.11.007

[3] Mahajan A . Proton craniospinal radiation therapy: rationale and clinical evidence. Int J Part Ther. 2014;1:399–407.

[4] Liebl J , Paganetti H , Zhu M , Winey BA . The influence of patient positioning uncertainties in proton radiotherapy on proton range and dose distributions. Med Phys. 2014;41:091711. 25186386 10.1118/1.4892601 PMC5148037

[5] Riboldi M , Orecchia R , Baroni G . Real‐time tumour tracking in particle therapy: technological developments and future perspectives. Lancet Oncol. 2012;13:e383–e391. 22935238 10.1016/S1470-2045(12)70243-7

[6] Fattori G , Riboldi M , Scifoni E , et al. Dosimetric effects of residual uncertainties in carbon ion treatment of head chordoma. Radiother Oncol. 2014;113:66–71. 25156944 10.1016/j.radonc.2014.08.001

[7] Paganetti H . Proton Therapy Physics (Series in Medical Physics and Biomedical Engineering). CRC Press; 2012 http://www.lavoisier.fr/livre/notice.asp?id=RK2W6SA2XKKOWQ.

[8] Coutrakon G , Bashkirov V , Hurley F , et al. Design and construction of the first proton CT scanner In: Application of Accelerators in Research and Industry. Melville, NY: AIP Publishing; 2013:327–331.

[9] Sadrozinski HF . Detector development for proton computed tomography (pCT) representing the pCT collaboration. In: IEEE Nuclear Science Symposium.; 2011:4457–4461.

[10] Johnson RP , Bashkirov V , Dewitt L , et al. A fast experimental scanner for proton CT: technical performance and first experience with phantom scans. IEEE Trans Nucl Sci. 2016;63:52–60. 27127307 10.1109/TNS.2015.2491918 PMC4844465

[11] Li T , Liang Z , Singanallur JV , Satogata TJ , Williams DC , Schulte RW . Reconstruction for proton computed tomography by tracing proton trajectories: a Monte Carlo study. Med Phys. 2006;33:699–706. 16878573 10.1118/1.2171507 PMC1550979

[12] Schulte RW , Penfold SN , Tafas JT , Schubert KE . A maximum likelihood proton path formalism for application in proton computed tomography. Med Phys. 2008;35:4849–4856. 19070218 10.1118/1.2986139

[13] Collins‐Fekete CA , Volz L , Portillo SK , Beaulieu L , Seco J . A theoretical framework to predict the most likely ion path in particle imaging. Phys Med Biol. 2017;62:1777. 28076336 10.1088/1361-6560/aa58ce

[14] Hurley RF , Schulte RW , Bashkirov VA , et al. Water‐equivalent path length calibration of a prototype proton CT scanner. Med Phys. 2012;39:2438–2446. 22559614 10.1118/1.3700173 PMC3338592

[15] Karonis NT , Duffin KL , Ordoñez CE , et al. Distributed and hardware accelerated computing for clinical medical imaging using proton computed tomography (pCT). J Parallel Distrib Comput. 2013;73:1605–1612.

[16] Penfold SN , Schulte RW , Censor Y , Rosenfeld AB . Total variation superiorization schemes in proton computed tomography image reconstruction. Med Phys. 2010;37:5887–5895. 21158301 10.1118/1.3504603 PMC2980547

[17] Yoo TS , Ackerman MJ , Lorensen WE , et al. Engineering and algorithm design for an image processing Api: a technical report on ITK–the Insight Toolkit. Stud Health Technol Inform. 2002;85:586–592. 15458157

[18] Mattes D , Haynor D . Nonrigid multimodality image registration. In: SPIE. Vol 4322.; 2001:1609–1620.

[19] Johnson HJ , Mccormick M , Ibanez L , Consortium IS . The ITK Software Guide Third Edition – Updated for ITK version 4.5. 2013 http://itk.org/ItkSoftwareGuide.pdf.

[20] Byrd RH , Lu P , Nocedal J , Zhu C . A limited memory algorithm for bound constrained optimization. SIAM J Sci Comput. 1995;16:1190–1208.

[21] McKay MD , Beckman RJ , Conover WJ . Comparison of three methods for selecting values of input variables in the analysis of output from a computer code. Technometrics. 1979;21:239–245.

[22] Cheung W , Hamarneh G . n‐SIFT: n‐Dimensional scale invariant feature transform. IEEE Trans Image Process. 2009;18:2012–2021. 19502129 10.1109/TIP.2009.2024578

[23] Paganelli C , Peroni M , Riboldi M , et al. Scale invariant feature transform in adaptive radiation therapy: a tool for deformable image registration assessment and re‐planning indication. Phys Med Biol. 2013;58:287–299. 23257263 10.1088/0031-9155/58/2/287

[24] Plautz TE , Bashkirov V , Giacometti V , et al. An evaluation of spatial resolution of a prototype proton CT scanner. Med Phys. 2016;43:6291–6300. 27908179 10.1118/1.4966028 PMC5097050

